# Genetic diversity and sex‐bias dispersal of plateau pika in Tibetan plateau

**DOI:** 10.1002/ece3.3289

**Published:** 2017-08-22

**Authors:** Liangzhi Zhang, Jiapeng Qu, Kexin Li, Wenjing Li, Min Yang, Yanming Zhang

**Affiliations:** ^1^ Key Laboratory of Adaptation and Evolution of Plateau Biota Northwest Institute of Plateau Biology, Chinese Academy of Sciences Xining Qinghai China; ^2^ Qinghai Key Laboratory of Animal Ecological Genomics Northwest Institute of Plateau Biology, Chinese Academy of Sciences Xining Qinghai China

**Keywords:** genetic diversity, microsatellite, plateau pika, population structure, sex‐bias dispersal

## Abstract

Dispersal is an important aspect in organism's life history which could influence the rate and outcome of evolution of organism. Plateau pika is the keystone species in community of grasslands in Tibetan Plateau. In this study, we combine genetic and field data to character the population genetic pattern and dispersal dynamics in plateau pika (*Ochotona curzoniae*). Totally, 1,352 individual samples were collected, and 10 microsatellite loci were analyzed. Results revealed that plateau pika possessed high genetic diversity and inbreeding coefficient in a fine‐scale population. Dispersal distance is short and restricted in about 20 m. An effective sex‐biased dispersal strategy is employed by plateau pika: males disperse in breeding period for mating while females do it after reproduction for offspring and resource. Inbreeding avoiding was shown as the common driving force of dispersal, together with the other two factors, environment and resource. In addition, natal dispersal is female biased. More detailed genetic analyzes are needed to confirm the role of inbreeding avoidance and resource competition as ultimate cause of dispersal patterns in plateau pika.

## 1 INTRODUCTION

Dispersal of individual is an important aspect in organism's life history which has profound significance for genetic structure, population biology and gene flow (Garant, Forde, & Hendry, 2007; Slatkin, 1987) that eventually could influence the rate and outcome of evolution of organism (Wright, 1931). The potential driving forces of dispersal mainly focused on several aspects including inbreeding, kin competition, resource competition, environment, and so on. Among them, inbreeding avoidance is recognized as the mainly cause of dispersal (Bowler & Benton, 2005).

In animals, sexual differences of dispersal are found and documented mainly on rates and distance. Dispersal rate is male biased in most of mammal species (Greenwood, 1980) and males also disperse in longer distances than females (Waser & Strobeck, 1998). Recently, some empirical studies have demonstrated the differences in dispersed distance between sexes (Blundell, Ben‐David, Groves, Bowyer, & Geffen, 2002; Fontanillas, Petit, & Perrin, 2004; Ji, Sarre, Aitken, Hankin, & Clout, 2001). The reasons of long‐ or short‐distance dispersal are likely to be very different (Ronce, Olivieri, Clobert, & Danchin, 2001). It has been evidenced that short‐distance dispersal is sufficient for avoiding inbreeding or kin competition, whereas long‐distance dispersal might function to colonize a new territory or escape (Perrin & Goudet, 2001). Dispersed distance is very informative for explicating the evolutionary cause of dispersal (Murrell, Travis, & Dytham, 2002). Furthermore, dispersed time might also be able to be another cause of dispersal. The timing of dispersal can be quite variable which depends on the species' life history (Schülke, 2003; Smale, Nunes, & Holekamp, 1997). Natal dispersal is thought to function as an effective means of spatially separating opposite‐sexed kin, alleviating the risk of close inbreeding (Dobson, 1982; Hazlitt, Eldridge, & Goldizen, 2004); while many species undergo secondary dispersal later in life, which is motivated for several purposes: such as aggressive eviction, and mating competition (Packer, 1979). In addition, dispersal increases with the social complexity (Perrin & Lehmann, 2001).

Plateau pika (*Ochotona curzoniae*) is small, diurnal, and nonhibernating rodent, belonging to lagomorph family, Ochotonidae. They mainly distribute within and around the Tibetan Plateau. They are highly social burrow dwellers with seasonally breeding (Dobson, Smith, & Wang, 1998; Lai & Smith, 2003; Smith & Foggin, 1999; Smith & Wang, 1991). They play pivotal role in the community dynamics of high‐altitude grasslands and are considered a keystone species in Tibetan Plateau.

With the development of statistical and genetic methods, many studies have performed spatial genetic structure analysis using genetic markers (Hazlitt et al., 2004; Peakall, Ruibal, & Lindenmayer, 2003). To date, most conclusions about genetic structure and dispersal of plateau pika are based on observational studies without full consideration of the spatial genetic structure of population (Dobson et al., 1998; Smith & Wang, 1991). In this study, we used a set of 10 microsatellite genetic markers to investigate the spatial genetic structure of plateau pika in a fine‐scale population, together with the observation data of field. The goals of this study are as follows: (i) dynamic of genetic diversity and population structure in plateau pika; (ii) sex‐biased dispersal pattern of plateau pika.

## 2 MATERIALS AND METHODS

### 2.1 Field description

**Figure 1:**
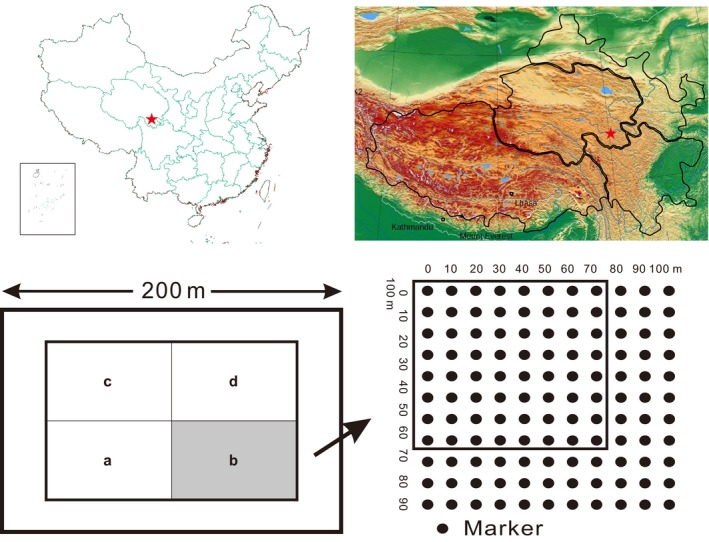
Sampling sites and the study area design

Plateau pika inhabits at the sample collection site, Maqin County (34°24′N, 100°21′E), Tibetan Autonomous Prefecture of Golog, Qinghai Province, People's Republic of China, where the average altitude is about 3,846 m (Figure 1). It is dry and cold, and the rainfall is mainly in summer (from June to August). The predominant vegetation of this area is kobresia humilis meadow. In the habitat, plateau pika is the most abundant rodent (Jiapeng et al., 2016).

### 2.2 Sample collection

Pikas were trapped using catch‐mark‐release method within a plot of 200 × 200 m region (the core region with 150 × 150, and 50 m around in distance as the relief area, Figure 1). We used toe clipping to mark pika and obtain tissues. Trapping is performed in May–August from 2005 to 2009 (Table S1). Body mass, age, time of recruitment, reproductive condition, and the relative coordinate of trapped sites were recorded. During breeding season, two groups could be recognized (i) mature one: overwintered adults who attend breeding; (ii) immature one: born in mating season, sexual immaturity who do not attend breeding (Table S1). All samples are carried out in strict accordance with the ethical guidelines approved by the Animal Care Commission of Plateau Biology, Chinese Academy of Sciences.

A total of 1,352 samples were trapped. Toe tissues are immediately preserved in 90% ethanol and then stored in 20°C.

### 2.3 DNA extraction and microsatellite analysis

Genomic DNA was extracted by standard phenol–chloroform extraction protocol and quantified by spectrophotometry and agarose gel electrophoresis. Characteristics of microsatellite markers are summarized in Table S2. All 10 microsatellite loci are amplified and genotyped basing on Kexin, Jianing, Jin, Yanming, and Songnian (2009).

### 2.4 Detection of genetic outliers

GeneClass2 was used to identify genetic outliers which might have been misidentified in the field (Piry et al., 2004). Using the frequencies, we calculated the log‐likelihood that an individual belongs to a population from a given year, and then we assessed the probability of an individual to be a resident using the Monte Carlo resampling algorithm (Peakall, Smouse, & Huff, 1995).

### 2.5 Genetic variability and genetic differentiation over time

Genetic variables in the five consecutive years were analyzed by CERVUS 3.0.3. Locus‐specific information was obtained, including number of alleles (Ne), observed heterozygosity (Ho), expected heterozygosity (He), allelic richness (AR), gene diversity (GD), polymorphism information content (PIC), and Hardy–Weinberg equilibrium (Marshall, Slate, Kruuk, & Pemberton, 1998).

### 2.6 The genetic structure of fine‐scale population

Population genetic structures were analyzed by STRUCTURE 2.3.4 (Evanno, Regnaut, & Goudet, 2005; Pritchard, Stephens, & Donnelly, 2000). We assumed an ancestry model: incorporating admixture, a model of correlated allele frequencies, without prior information corresponding to the origin of samples. Ten independent runs of *K* = 1–30 were performed at 100,000 Markov Chain Monte Carlo (MCMC) repetitions and a 100,000 burn‐in period. The most likely *K* was identified by web‐based tool STRUCTURE HARVESTER v0.6.8 (Earl & Von‐Holdt, 2011).

### 2.7 Assignment tests and relatedness

Assignment tests were executed to evaluate the sex‐bias dispersal of population via the program FSATAT version 2.9.3.2 (Goudet, 2002). Genetic variance among populations (*F*_ST_), mean/variance of assignment index (mAIc/vAIc), and the average relatedness of individuals within a population relative to the whole sample (*r*,* r* = 2*F*_ST_/1+*F*_IT_) were analyzed for both sexes using 10,000 permutations in the menu of biased dispersal.

### 2.8 Spatial genetic structure and sex‐biased dispersal analysis

Program GenAlEx 6.5 was used to calculate the matrices of pairwise genetic distances among groups (Peakall & Smouse, 2006). Multilocus spatial autocorrelation analysis was proven powerful and flexible for detecting fine‐scale genotypic structure in animals (Peakall et al., 2003; Smouse & Peakall, 1999; Smouse, Peakall, & Gonzales, 2008). This method does not need to make assumptions about the relationship between geographic and genetic distances. Banks and Peakall (2012) described a spatially explicit individual‐based simulation framework to investigate the effects of sex‐specific variation in dispersal on fine‐scale genetic structure (Banks & Peakall, 2012). In this study, we used this method to analyze the dispersed pattern of pika. Briefly, there were three steps. First, we estimated the bootstrap 95% confidence intervals about *r* by drawing with replacement within the set of relevant pairwise comparisons for a given distance class. Second, the standard spatial autocorrelation analysis of sexes was performed in separate populations, and then the nonparametric heterogeneity tests were computed (Smouse et al., 2008). Finally, the two correlograms drawn on the *p* values for the T2 statistic at each distance class across both populations were compared, and the correlograms wide “Omega” (ω) were computed. In our study, the distance class is 10 m (first distance class is 0–10 m). The first distance class was determined based on the family zone of plateau pika from previous study and our observation of field (Dobson et al., 1998). Data from each sampling period were explored at geographic distance intervals ranging from 10 to 200 m and 10 m intervals.

## 3 RESULTS

### 3.1 Population genetic diversity and inbreeding coefficient in five consecutive years

Genetic diversities in five consecutive years are listed in Table 1. We find high genetic variability in the population: AR varies between 8.21 and 10.87 (Table S4); expected heterozygosity (He) is about 0.70 in 5 years; the mean effective number of alleles (Ne) is among 13.7 to 14.9; GD is about 0.8 (Table S3). The PIC ranges from 0.77 to 0.82, which indicate high genetic diversity in populations (PIC value < 0.25, low polymorphism; 0.25 < PIC value < 0.5, intermediate polymorphism; and PIC value > 0.5, high polymorphism). Mean inbreeding coefficient (*F*_IS_) ranges from 0.11 to 0.21 among 5 years (Table S5), which implies that the nonrandom mating occurred in populations.

**Table 1:** Summary of genetic variation characteristics for 10 microsatellite loci in this study

Years	*N*	Typed	Ne	Ho	He	PIC	AR	GD	*F*_IS_	*F*_ST_	*R*	*K*
2005	306	0.88	17.40	0.70	0.82	0.80	10.87	0.83	0.15	0.0015	0.003	22
2006	219	0.92	13.70	0.71	0.80	0.77	9.26	0.80	0.11	0.002	0.004	23
2007	160	0.87	14.50	0.70	0.83	0.81	8.21	0.83	0.16	0.0024	0.004	22
2008	352	0.85	16.70	0.71	0.83	0.81	10.85	0.84	0.15	0.0032	0.006	22
2009	315	0.86	16.90	0.66	0.84	0.82	10.06	0.84	0.21	−0.0045	−0.002	28

*N*, genotyped individuals; Typed, mean number of loci typed; Ne, effective number of alleles; Ho, observed heterozygosity; He, expected heterozygosity; PIC, polymorphism information content; AR, allelic richness; GD, gene diversity; *F*_IS_, fixation index; *R*, relatedness of individuals; *K*, number of groups in the population.

### 3.2 Population structure in five consecutive years

We identified genetic clusters following the procedure described by Evanno et al. (2005). In independent simulations of structure analysis, a distinct apex value of DK is at K (Fig. S1). The most probable number of genetic clusters in different years is listed in Table 1. According to the rule of STRUCTURE HARVESTER, the most genetic clusters are 2 in 2005 (*K* = 2), but it is in contrary with the observation in the field. Considering the result of observation, the clusters are defined 22 (*K* = 22).

### 3.3 Assignment tests and sex differences in relatedness

The assignment test is applied to analyze the sex‐biased dispersal in population level. Result suggests that there is no significant difference between opposite sexes in estimates of population structure (*F*_ST_ and *r*), assignment index (mAIc and vAIc), or inbreeding index (*F*_IS_; Table 2), except for that in 2008. The mAIc is negative for males and positive for females in all years (Table 2). *F*_ST_, *r* and mAIc are expected to be higher in philopatric ones than that of dispersed ones. Immigrants tend to have lower AIc values than residents (Goudet, Perrin, & Waser, 2002). In our population, females have greater probability of being residents because the mAIC of females is positive while the male is negative. And in 2008, mAIc and vAIc are significantly higher in males than females (both *p* < .05, Table 2), it is consistent with male‐biased dispersal. But the number of female is much larger than that of male in 2008, which maybe resulted into the difference of AIc.

**Table 2:** The sex‐biased dispersal analysis in population level

	Sex	*N*	*F*_IS_	*F*_ST_	*r*	mAIc	vAIc
2005	Female	176	0.1449	0.0033	0.0057	0.12448	34.28819
	Male	130	0.1566	0.0023	0.004	−0.16852	25.8334
	Overall	306	0.1508	0.0015	0.0026	NA	NA
	*p*‐value	NA	.37	.32	.33	.25	.97
2006	Female	125	0.11	0.002	0.0036	0.22069	18.86144
	Male	94	0.1077	0.0022	0.004	−0.29347	18.1445
	Overall	219	0.1091	0.002	0.0037	NA	NA
	*p*‐value	NA	.4	.45	.45	.2	.65
2007	Female	74	0.1632	−0.0002	−0.0003	0.43665	13.02787
	Male	86	0.1614	0.0031	0.0053	−0.37572	14.19783
	Overall	NA	0.1615	0.0024	0.0041	NA	NA
	*p*‐value	NA	.58	.74		.08	.21
2008	Female	224	0.1391	0.0044	0.0078	0.43142	15.64061
	Male	128	0.1672	0.0062	0.0106	−0.75499	22.28215
	Overall	352	0.1505	0.0032	0.0055	NA	NA
	*p*‐value	NA	.1	.81	.77	.01	.03
2009	Female	131	0.2327	0.0001	0.0002	0.05884	13.80858
	Male	184	0.1923	−0.0027	−0.0045	−0.08264	17.00434
	Overall	315	0.2092	−0.001	−0.0016	NA	NA
	*p*‐value	NA	.98	.17	.17	.62	.84

*F*_IS_, the level of inbreeding within a population relative to the whole sample; *F*_ST_, the proportion of genetic variation among populations; *r*, the average relatedness of individuals within a population relative to the whole sample (where *r* = 2*F*_ST_/1+*F*_IT_); mAIc, the mean of the assignment index; vAIc, the variance of the assignment index. The *p* values are estimated using 10,000 randomizations.

### 3.4 Spatial genetic structure of plateau pika population

In the study, individuals are divided into two groups as described above. Individuals from each period were analyzed in mature group. Mature females are genetically similar to local ones rather than distant ones in May to July: pairs exhibit significant positive spatial genetic structure at 0–20 m, while they are more genetically similar to geographically distant ones in August (Figure 1a). But the mature males are in contrary: more genetically similar to geographically distant males in May to July; pairs exhibited almost no positive spatial genetic structure at 0–20 m, while they are more genetically similar to local distant ones in August (Figure 1b). The difference in spatial genetic structures between sexes is quite distinct at 0–20 m.

In immature group, females are genetically similar to local ones rather than distant ones in May and June: pairs exhibited significant positive spatial genetic structure at 0–50 m, while they are more genetically similar to geographically distant ones in July and August (Figure 1c). However, the males exhibit genetically similar to local ones in whole periods (Figure 1d).

### 3.5 Sex‐biased dispersal based on the genetic spatial autocorrelation

Spatial analysis of multilocus is a powerful approach for detecting sex‐biased dispersal in natural populations (Banks & Peakall, 2012). Three statistical tests were used: bootstrap confidence intervals about the autocorrelation *r* values and two recently developed heterogeneity tests at the distance class. And whole correlogram levels have been used to identify the sex‐biased dispersal.

**Figure 2:**
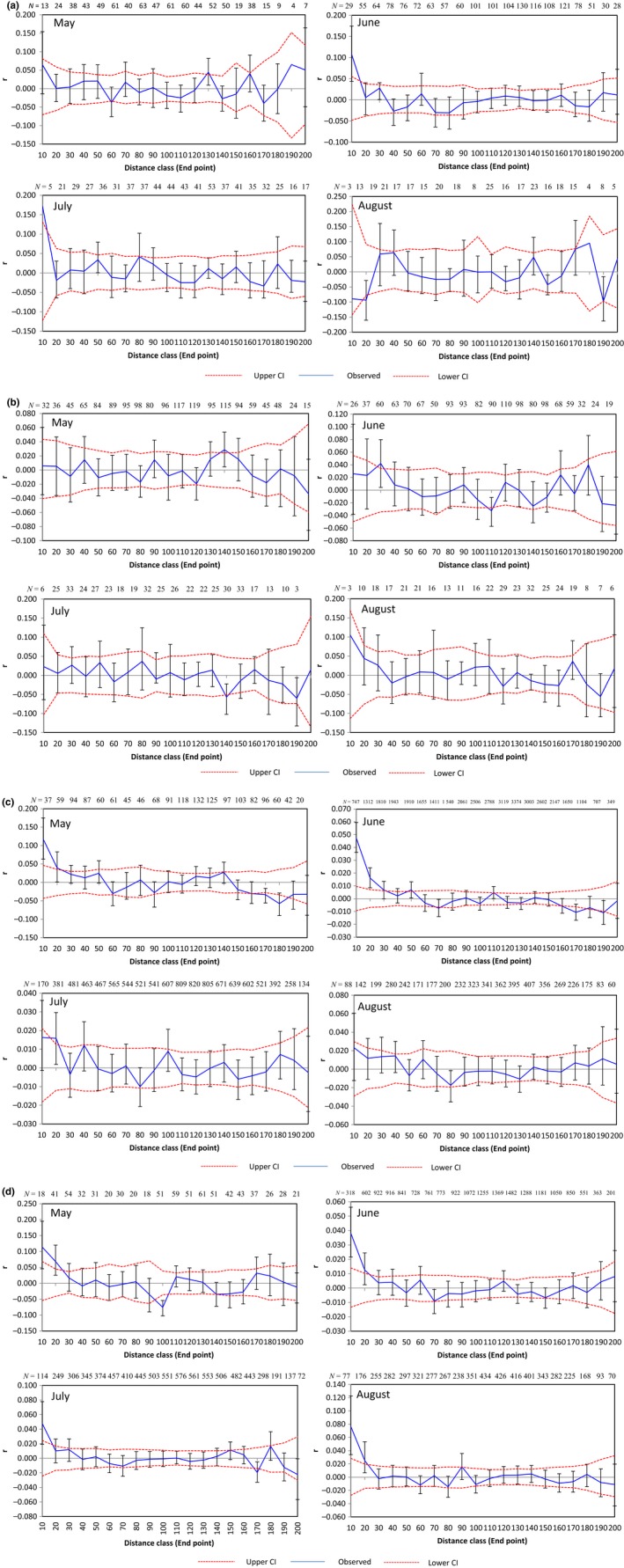
Spatial genetic structure correlograms for different pika population at study area from May to August. The sample size at each distance class is provided above each correlogram. The scale for the autocorrelation coefficients is not standardized because of the inherent skew in relatedness between the sexes. (a) Spatial genetic structure correlograms for mature female pika population; (b) Spatial genetic structure correlograms for mature male pika population; (c) Spatial genetic structure correlograms for immature female pika population; (d) Spatial genetic structure correlograms for immature male pika population

For maturity group, it showed that female pairs have more pronounced genetic structure than male pairs at the shortest distance class (especial in the first class), as indicated by significantly lower spatial autocorrelation coefficients of males in May to July, while the result is opposite in August. The result indicates that male dispersed while female was more philopatric in mating period from May to July, but the situation reversed in the end of breeding in August (Figures 2 and 4).

**Figure 3:**
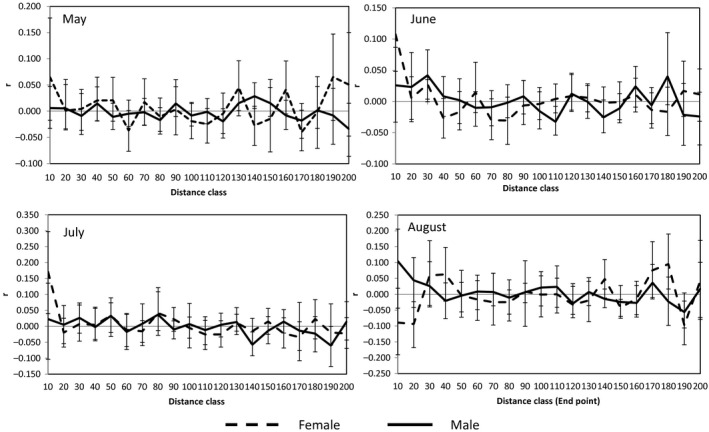
Autocorrelation (“relationship”) coefficients (*r*) of each mature sex with geographic distance (the interval of each distance class is 10 m) with significance indicated by nonoverlapping bootstrap error bars. The error bars represent bootstrap 95% confidence intervals around each autocorrelation coefficient (*r*)

**Figure 4:**
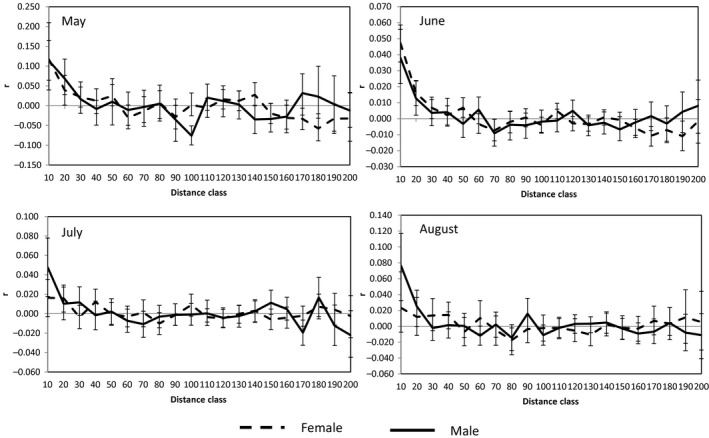
Autocorrelation (“relationship”) coefficients (*r*) of immature sex with geographic distance (the interval of each distance class is 10 m) with significance indicated by nonoverlapping bootstrap error bars. The error bars represent bootstrap 95% confidence intervals around each autocorrelation coefficient (*r*)

For immaturity group, both sexes displayed higher spatial autocorrelation coefficients in May and June, while male was with much higher than female in July and August (Figures 3 and 4). It indicates that the immature males were more philopatric in whole period, and immature females dispersed in July and August. The immature females disperse in July when they reach body maturity and the behavior will continue until August when all females finish the dispersal.

## 4 DISCUSSION

### 4.1 Genetic diversity and population structure

Genetic diversity is an important aspect of population dynamics which directly relates with evolutionary potential and inbreeding (Hughes, Inouye, Johnson, Underwood, & Vellend, 2008). In our research, plateau pika possesses high genetic diversity (Table 1 and Tables S3–S5). It was evidenced that decrease of observed heterozygosity (Ho) in population could induce the decrease in average fitness of individuals (Reed & Frankham, 2003; Szulkin, Bierne, & David, 2010), so high Ho may indicate great potential fitness of individual. Furthermore, AR is closely related to the adaptive ability of population for environmental changes, and allelic richness is considered as a strong indicator of the evolutionary potential of population (Allendorf, 1986; Allendorf, Luikart, & Aitken, 2012; Caballero & García‐Dorado, 2013). Genetic diversity of plateau pika is much more abundant than others. American pika is characterized by low levels of allelic richness (2.77) and observed heterozygosity (0.39). It has reported that genetic diversity decreases as population density declines (Jiayan & Zhibin, 2006). American pika population decline obviously, which is considered as an endangered species (Gibson, Van Der Marel, & Starzomski, 2009; Kelsey, Clayton, & Michael, 2016). Collared pika, which lives primarily on talus, showed a medium genetic diversity (Jessie & David, 2012). We compared several species and found the genetic diversity reduces with the lowing of elevation (Table S6). It indicates that pika is more suitable for high elevation environment. The divergence in genetic diversity of species may be caused by ecological disturbances. Ecological disturbance is considered as a driver of genetic diversity. Disturbance may cause a loss of genetic diversity within population (in particular, allelic diversity or richness) when population sizes were reduced (Banks, Blyton, Blair, McBurney, & Lindenmayer, 2012).

Furthermore, the inbreeding coefficient of plateau pika is medium in three species (Table 2 and Table S6), but it is still harmful for the stabilization of population. Inbreeding depression could reduce population performance through increasing the recessive or deleterious traits of offspring (Banks et al., 2012). Inbreeding depression is closely related to the evolutionary of animal (Charlesworth & Willis, 2009). For plateau pika, the *F*_IS_ value showed that inbreeding indeed existed in population (Table 2). But this is contradicted with the high genetic diversity in some extent. Inbreeding coefficient is different in three pika species (Table S6). The result show that inbreeding is severe in American pika (Kelsey, Clayton, & Michael, 2016), and then in plateau pika. However, there is almost no inbreeding in collared pika population. This may be related to their life history and dispersal patterns (Lawson Handley & Perrin, 2007). In addition, population size is another factor (Dlugosch & Parker, 2008). American pika reduced rapidly, which may be cause by serious inbreeding in ever‐reduced population.

### 4.2 Characteristic of dispersal in plateau pika population

Among animals, dispersal pattern is different between sexes in terms of distances or rates. It reported that plateau pika was philopatric, and dispersal distance was extremely restricted. Furthermore, they dispersed often among families (Dobson et al., 1998). Our results showed that dispersal distance of plateau pika was very limited, within 20 m, which is consistent with the previous observation of 1.3 family ranges (average distance between centers of activity within families is 5.7 m and average distance separating the centers of activity of neighboring families is 23.8 m; Dobson et al., 1998). The restricted dispersal has also been demonstrated in American pika (Kelsey et al., 2016). It reported that short‐distance dispersal was probably sufficient for avoiding inbreeding or kin competition (Perrin & Goudet, 2001).

In our study, the male‐biased dispersal is not obvious in pika population. Since warm season is very short in Tibetan Plateau, resource (especially food) is considered as a great limiting factor for performance of alpine biota. So in this analysis, dispersal time has been in full consideration. The timing of dispersal could provide interesting clues to ultimate pattern. Natal dispersal was thought of a fundamental parameter in life history, and population biology (Garant et al., 2007; Slatkin, 1987). And natal dispersal was the most common mechanisms proposed to reduce the risk of inbreeding (Lawson Handley & Perrin, 2007; Perrin & Mazalov, 1999). In this study, the natal dispersal of plateau pika is female biased: female juveniles dispersed while the male juveniles were philopatric, which is uncommon in small mammals. We also saw the second dispersal of pika in the mating period. The mature male dispersed in mating period while female dispersed when they finished reproduction (Figures 2 and 4; Table 3).

**Table 3:** Dispersed characteristic of plateau pika

Age	Dispersed sex	Dispersed time	Dispersed purpose	Dispersed name
Maturity	Male	May, June, July	Mating, inbreeding avoiding	Second dispersal
	Female	August	Inbreeding avoiding, resource	
Immaturity	Female	July, August	Inbreeding avoiding, resource	Natal dispersal

The extraordinary dispersal pattern of plateau pika can explain the contrary between genetic diversity and inbreeding coefficient. Dispersal strategies evolve a large variety of reasons. For mature male, the dispersal in mating period may drive by two factors: inbreeding avoidance, which was the common driving force in mammal (Charlesworth & Willis, 2009); and local mate competition, because the survival rate can be almost lower than 50% in small mammals, and the “mortality costs” require high reproductive capacity, so adults need more chance to mate (Johnson & Crossman, 1991). The short‐distance dispersal is an effective way for male to get more mating chance with limited costs.

For female (including juvenile and maturity), the dispersal is also related to breeding avoiding, that is reducing the risk of father–daughter mating (Lawson Handley & Perrin, 2007), and resource (Greenwood, 1980). In Tibetan plateau, the environment is harsh; resource is limited for survival, especially in winter. The cost of immigration must be in full of consideration. Female dispersal may be a better choice with less cost; they could be more readily accepted into a new group for social animals. Furthermore, postweaning territory is inheritable, where juveniles (especially for male) need to acquire resource such as a burrow to survive in severe environment (Price & Boutin, 1993). The main reasons of distinctive dispersal pattern for pika are inbreeding avoiding and resource.

The dispersal strategy of plateau is effective, but there are still some defects. Firstly, it is cannot completely avoid inbreeding (Table 2). This may be caused by restricted dispersal. Although parallel dispersal between sexes can reduce the inbreeding greatly, in the confined spaces the related individuals also can mate. Moreover, the short life span can also reduce the inbreeding in some extend. However, the strategy is still successful for plateau pika population.

## CONFLICT OF INTEREST

None declared.

## References

[ece33289-bib-0001] Allendorf, F. W. (1986). Genetic drift and the loss of alleles versus heterozygosity. Zoo Biology, 5(2), 181–190.

[ece33289-bib-0002] Allendorf, F. W. , Luikart, G. L. , & Aitken, S. N. (2012). Conservation and the genetics of populations: Loss of genetic variation: The inbreeding effect of small populations. Oxford, UK: Wiley‐Blackwell Press.

[ece33289-bib-0003] Banks, S. C. , Blyton, M. D. J. , Blair, D. , McBurney, L. , & Lindenmayer, D. B. (2012). Adaptive responses and disruptive effects: How major wildfire influences kinship‐based social interactions in a forest marsupial. Molecular Ecology, 21(3), 673–684. 21929555 10.1111/j.1365-294X.2011.05282.x

[ece33289-bib-0004] Banks, S. , & Peakall, R. (2012). Genetic spatial autocorrelation can readily detect sex‐biased dispersal. Molecular Ecology, 21(9), 2092–2105. 22335562 10.1111/j.1365-294X.2012.05485.x

[ece33289-bib-0005] Blundell, G. M. , Ben‐David, M. , Groves, P. , Bowyer, R. T. , & Geffen, E. (2002). Characteristics of sex‐biased dispersal and gene flow in coastal river otters: Implications for natural recolonization of extirpated populations. Molecular Ecology, 11(3), 289–303. 11928704 10.1046/j.0962-1083.2001.01440.x

[ece33289-bib-0006] Bowler, D. E. , & Benton, T. G. (2005). Causes and consequences of animal dispersal strategies: Relating individual behaviour to spatial dynamics. Biology Review of the Cambridge Philosophical Society, 80(2), 205–225. 10.1017/s1464793104006645 15921049

[ece33289-bib-0007] Caballero, A. , & García‐Dorado, A. (2013). Allelic diversity and its implications for the rate of adaptation. Genetics, 195(4), 1373–1384. 24121776 10.1534/genetics.113.158410 PMC3832279

[ece33289-bib-0008] Charlesworth, D. , & Willis, J. H. (2009). FUNDAMENTAL CONCEPTS IN GENETICS: The genetics of inbreeding depression. Nature Reviews Genetics, 10(11), 783–796. 10.1038/nrg2664 19834483

[ece33289-bib-0009] Dlugosch, K. M. , & Parker, I. M. (2008). Founding events in species invasions: Genetic variation, adaptive evolution, and the role of multiple introductions. Molecular Ecology, 17(1), 431–449. 17908213 10.1111/j.1365-294X.2007.03538.x

[ece33289-bib-0010] Dobson, F. S. (1982). Competition for mates and predominant juvenile male dispersal in mammals. Animal Behaviour, 30(11), 1183–1192.

[ece33289-bib-0011] Dobson, F. S. , Smith, A. T. , & Wang, X. G. (1998). Social and ecological influences on dispersal and philopatry in the plateau pika (*Ochotona curzoniae*). Behavioral Ecology, 9(6), 622–635.

[ece33289-bib-0012] Earl, D. A. , & Von‐Holdt, B. M. (2011). STRUCTURE HARVESTER: A website and program for visualizing STRUCTURE output and implementing the Evanno method. Conservation Genetics Resources, 4(2), 359–361.

[ece33289-bib-0013] Evanno, G. , Regnaut, S. , & Goudet, J. (2005). Detecting the number of clusters of individuals using the software STRUCTURE: A simulation study. Molecular Ecology, 14(8), 2611–2620. 15969739 10.1111/j.1365-294X.2005.02553.x

[ece33289-bib-0014] Fontanillas, P. , Petit, E. , & Perrin, N. (2004). Estimating sex‐specific dispersal rates with autosomal markers in hierarchically structured populations. Evolution, 58(4), 886–894. 15154563 10.1111/j.0014-3820.2004.tb00420.x

[ece33289-bib-0015] Garant, D. , Forde, S. E. , & Hendry, A. P. (2007). The multifarious effects of dispersal and gene flow on contemporary adaptation. Functional Ecology, 21(3), 434–443.

[ece33289-bib-0016] Gibson, S. , Van Der Marel, R. , & Starzomski, B. (2009). Climate change and conservation of leading‐edge peripheral populations. Conservation Biology, 23(6), 1369–1373. 20078636 10.1111/j.1523-1739.2009.01375.x

[ece33289-bib-0017] Goudet, J. (2002). FSTAT: a program to estimate and test gene diversities and fixation indices, Version 2.9.3.2. Retrieved from https://www2.unil.ch/popgen/softwares/fstat.htm

[ece33289-bib-0018] Goudet, J. , Perrin, N. , & Waser, P. (2002). Tests for sex‐biased dispersal using bi‐parentally inherited genetic markers. Molecular Ecology, 11(6), 1103–1114. 12030985 10.1046/j.1365-294x.2002.01496.x

[ece33289-bib-0019] Greenwood, P. J. (1980). Mating systems, philopatry and dispersal in birds and mammals. Animal Behaviour, 28(11), 1140–1162.

[ece33289-bib-0020] Hazlitt, S. L. , Eldridge, M. D. B. , & Goldizen, A. W. (2004). Fine‐scale spatial genetic correlation analyses reveal strong female philopatry within a brush‐tailed rock‐wallaby colony in southeast Queensland. Molecular Ecology, 13(2), 3621–3632. 15548278 10.1111/j.1365-294X.2004.02342.x

[ece33289-bib-0021] Hughes, R. A. , Inouye, B. D. , Johnson, M. T. J. , Underwood, N. , & Vellend, M. (2008). Ecological consequences of genetic diversity. Ecology Letter, 11(6), 609–623. 10.1111/j.1461-0248.2008.01179.x 18400018

[ece33289-bib-0022] Jessie, M. Z. , & David, S. H. (2012). Polygynandry and even‐sexed dispersal in a population of collared pikas, *Ochotona collaris* . Animal Behaviour, 83(4), 1075–1082.

[ece33289-bib-0023] Ji, W. , Sarre, S. D. , Aitken, N. , Hankin, R. K. S. , & Clout, M. N. (2001). Sex‐biased dispersal and a density‐independent mating system in the Australian brushtail possum, as revealed by minisatellite DNA profiling. Molecular Ecology, 10(6), 1527–1537. 11412373 10.1046/j.1365-294x.2001.01287.x

[ece33289-bib-0500] Jiapeng, Q. , Min, Y. , Wenjing, Li. , Qianquan, Chen. , Zhaorong, Mi. , Weixin, Xu. , et. al. (2016). Effects of climate change on the reproduction and offspring sex ratio of plateau pika (Ochotona curzoniae) on the Tibetan Plateau. Journal of Arid Environments, 134, 66–72.

[ece33289-bib-0024] Jiayan, X. , & Zhibin, Z. (2006). Genetic diversity decreases as population density declines: Implications of temporal variation in mitochondrial haplotype frequencies in a natural population of *Tscherskia triton* . Integrative Zoology, 1(4), 188–193. 21396012 10.1111/j.1749-4877.2006.00035.x

[ece33289-bib-0025] Johnson, C. N. , & Crossman, D. G. (1991). Dispersal and social organization of the Northern hairy‐nosed wombat *Lasiorhinus krefftii* . Journal of Zoology, 225(4), 605–613.

[ece33289-bib-0026] Kelsey, M. R. , Clayton, T. L. , & Michael, A. R. (2016). Low genetic diversity, restricted dispersal, and elevation specific patterns of population decline in American pikas in an atypical environment. Journal of Mammalogy, 97(2), 464–472.

[ece33289-bib-0027] Kexin, L. , Jianing, G. , Jin, Y. , Yanming, Z. , & Songnian, H. (2009). Isolation and characterization of 13 microsatellite loci in the plateau pika (*Ochotona curzoniae*). Conversion Genetics, 10(3), 785–787.

[ece33289-bib-0028] Lai, C. H. , & Smith, A. T. (2003). Keystone status of plateau pikas (*Ochotona curzoniae*): Effect of control on biodiversity of native birds. Biodiversity Conversion, 12(9), 1901–1912.

[ece33289-bib-0029] Lawson Handley, L. J. , & Perrin, N. (2007). Advances in our understanding of mammalian sex‐biased dispersal. Molecular Ecology, 16(8), 1559–1578. 17402974 10.1111/j.1365-294X.2006.03152.x

[ece33289-bib-0030] Marshall, T. C. , Slate, J. , Kruuk, L. E. B. , & Pemberton, J. M. (1998). Statistical confidence for likelihood‐based paternity inference in natural populations. Molecular Ecology, 7(5), 639–655. 9633105 10.1046/j.1365-294x.1998.00374.x

[ece33289-bib-0031] Murrell, D. J. , Travis, J. M. J. , & Dytham, C. (2002). The evolution of dispersal distance in spatially‐structured populations. Oikos, 97(2), 229–236.

[ece33289-bib-0032] Packer, C. (1979). Inter‐troop transfer and inbreeding avoidance in *Papio anubis* . Animal Behaviour, 27(2), 1–36. 10.1016/0003-3472(79)90127-1 555842

[ece33289-bib-0033] Peakall, R. , Ruibal, M. , & Lindenmayer, D. B. (2003). Spatial autocorrelation analysis offers new insights into gene flow in the Australian bush rat, *Rattus fuscipes* . Evolution, 57(5), 1182–1195. 12836834 10.1111/j.0014-3820.2003.tb00327.x

[ece33289-bib-0034] Peakall, R. , & Smouse, P. E. (2006). GENALEX 6: Genetic analysis in Excel. Population genetic software for teaching and research. Molecular Ecology Notes, 6(1), 288–295. 10.1093/bioinformatics/bts460 PMC3463245 22820204

[ece33289-bib-0035] Peakall, R. , Smouse, P. E. , & Huff, D. R. (1995). Evolutionary implications of allozyme and RAPD variation in diploid populations of dioecious buffalograss (*Buchloë dactyloides*). Molecular Ecology, 4(2), 135–147.

[ece33289-bib-0036] Perrin, N. , & Goudet, J. (2001). Inbreeding, kinship and the evolution of natal dispersal In J Clobert, E Danchin, A.A Dhondt, & J.D Nichols (Ed), Dispersal (pp. 123–142). Oxford, UK: Oxford University Press.

[ece33289-bib-0037] Perrin, N. , & Lehmann, L. (2001). Is sociality driven by the costs of dispersal or the benefits of philopatry? A role for kin‐discrimination mechanisms. American Naturalist, 158(5), 471–483. 10.1086/323114 18707302

[ece33289-bib-0038] Perrin, N. , & Mazalov, V. (1999). Dispersal and inbreeding avoidance. The American Naturalist, 154(3), 282–292. 10.1086/303236 10506544

[ece33289-bib-0039] Piry, S. , Alapetite, A. , Cornuet, J. M. , Paetkau, D. , Baudouin, L. , & Estoup, A. (2004). GeneClass2: A software for genetic assignment and first‐generation migrant detection. Journal of Heredity, 95(6), 536–539. 15475402 10.1093/jhered/esh074

[ece33289-bib-0040] Price, K. , & Boutin, S. (1993). Territory bequeathal by red squirrel mothers. Behavioral Ecology, 4(2), 144–150.

[ece33289-bib-0041] Pritchard, J. , Stephens, M. , & Donnelly, P. (2000). Inference of population structure using multilocus genotype data. Genetics, 155(2), 945–959. 10835412 10.1093/genetics/155.2.945 PMC1461096

[ece33289-bib-0043] Reed, D. H. , & Frankham, R. (2003). Correlation between fitness and genetic diversity. Conservation Biology, 17(1), 230–237.

[ece33289-bib-0044] Ronce, O. , Olivieri, I. , Clobert, J. , & Danchin, E. (2001). Perspectives on the study of dispersal evolution In J Clobert (Ed.), Dispersal (pp. 314–357). Oxford, UK: Oxford University Press.

[ece33289-bib-0045] Schülke, O. (2003). To breed or not to breed‐food competition and other factors involved in female breeding decisions in the pair‐living nocturnal fork‐marked lemur (*Phaner furcifer*). Behavioral Ecology and Sociobiology, 55(1), 11–21.

[ece33289-bib-0046] Slatkin, M. (1987). Gene flow and the geographic structure of natural populations. Science, 236(4803), 787–792. 3576198 10.1126/science.3576198

[ece33289-bib-0047] Smale, L. , Nunes, S. , & Holekamp, K. E. (1997). Sexually dimorphic dispersal in mammals: Patterns, causes and consequences. Advances in the Study of Behavior, 26, 181–250.

[ece33289-bib-0048] Smith, A. T. , & Foggin, J. M. T. (1999). Plateau pika (*Ochotona curzoniae*) is a keystone species for biodiversity on the Tibetan plateau. Animal Conservation, 2(4), 235–240.

[ece33289-bib-0049] Smith, A. T. , & Wang, X. G. (1991). Social relationships of adult black‐lipped pikas (*Ochotona curzoniae*). Journal of Mammalogy, 72(2), 231–247.

[ece33289-bib-0050] Smouse, P. E. , & Peakall, R. (1999). Spatial autocorrelation analysis of individual multiallele and multilocus genetic structure. Heredity, 82(5), 561–573. 10383677 10.1038/sj.hdy.6885180

[ece33289-bib-0051] Smouse, P. E. , Peakall, R. , & Gonzales, E. (2008). A heterogeneity test for fine‐scale genetic structure. Molecular Ecology, 17(14), 3389–3400. 18677808 10.1111/j.1365-294x.2008.03839.x

[ece33289-bib-0052] Szulkin, M. , Bierne, N. , & David, P. (2010). Heterozygosity‐fitness correlations: A time for reappraisal. Evolution, 64(5), 1202–1217. 20148954 10.1111/j.1558-5646.2010.00966.x

[ece33289-bib-0053] Waser, P. M. , & Strobeck, C. (1998). Genetic signatures of interpopulation dispersal. Trends in Ecology and Evolution, 13(2), 43–44. 21238195 10.1016/s0169-5347(97)01255-x

[ece33289-bib-0054] Wright, S. (1931). Evolution in Mendelian populations. Genetics, 16(2), 97–159. 17246615 10.1093/genetics/16.2.97 PMC1201091

