# Supplementary material for: Genetic diversity and sex‐bias dispersal of plateau pika in Tibetan plateau
Source: Ecol Evol. 2017 Aug 22;7(19):7708–18. doi: 10.1002/ece3.3289 (PMC5632614; doi:10.1002/ece3.3289)

FIGURE S1a: Detection of the most likely number of genetically distinct groups in 2005.

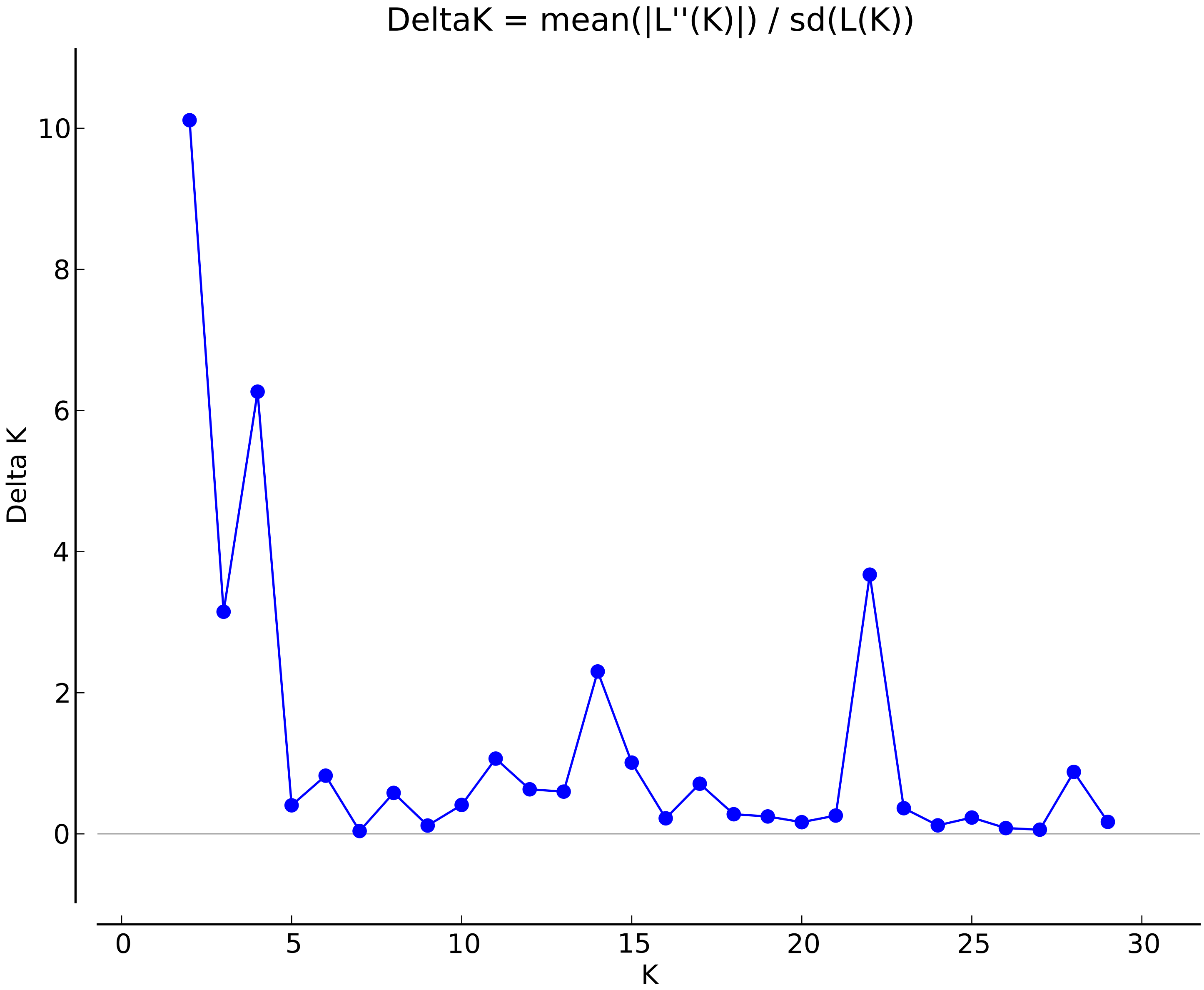

FIGURE S1b: Detection of the most likely number of genetically distinct groups in 2006.

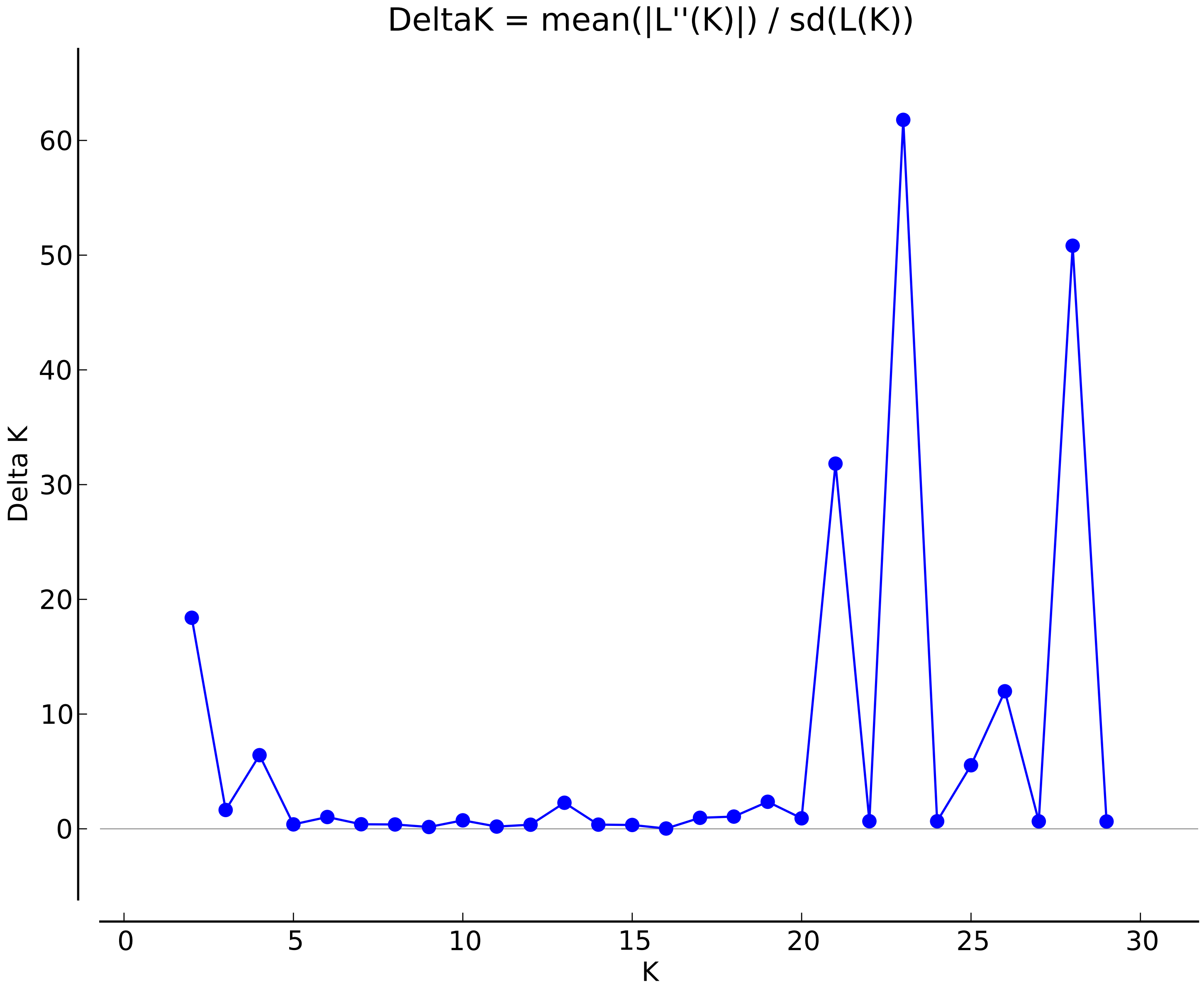

FIGURE S1c: Detection of the most likely number of genetically distinct groups in 2007.

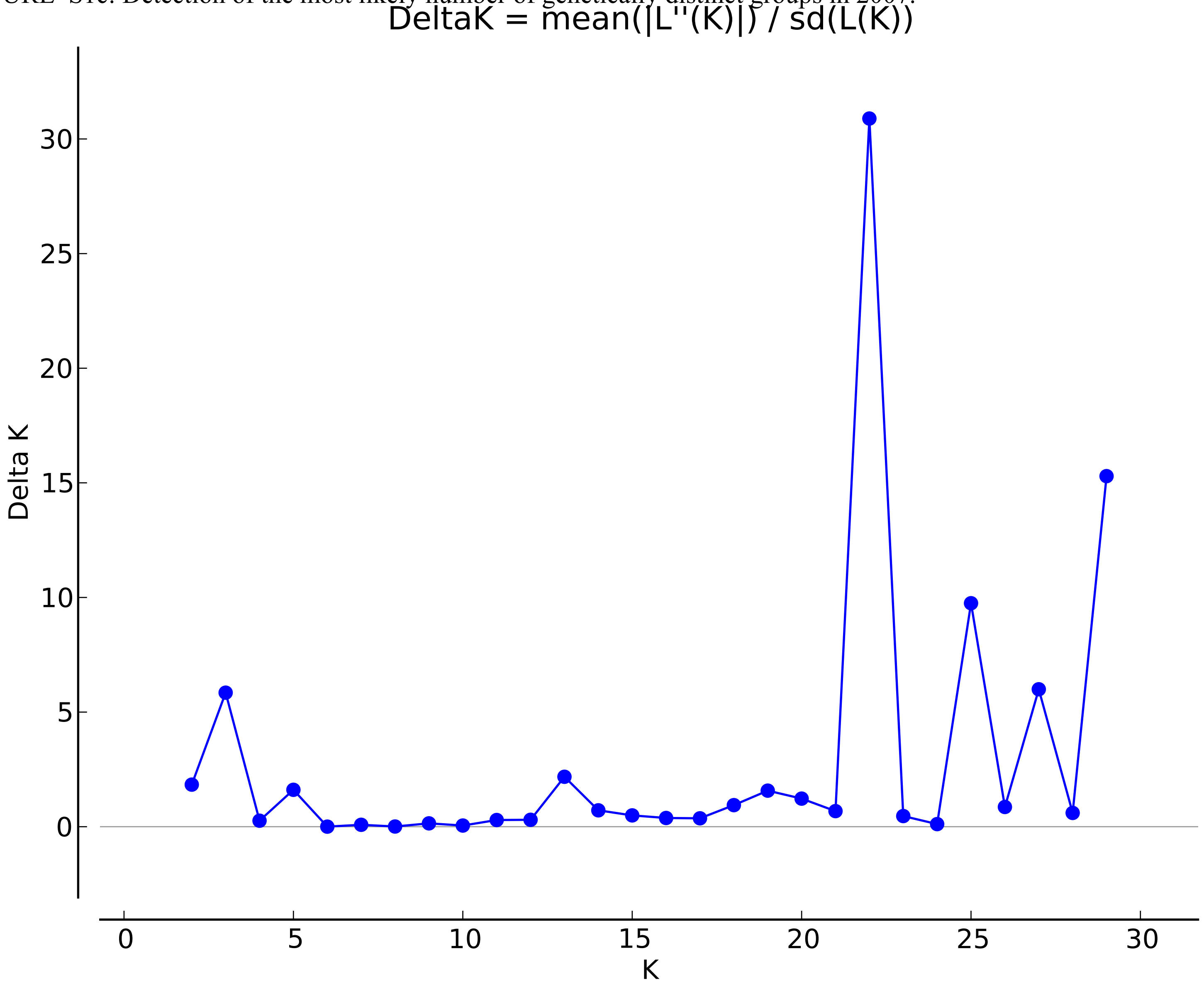

FIGURE S1d: Detection of the most likely number of genetically distinct groups in 2008.

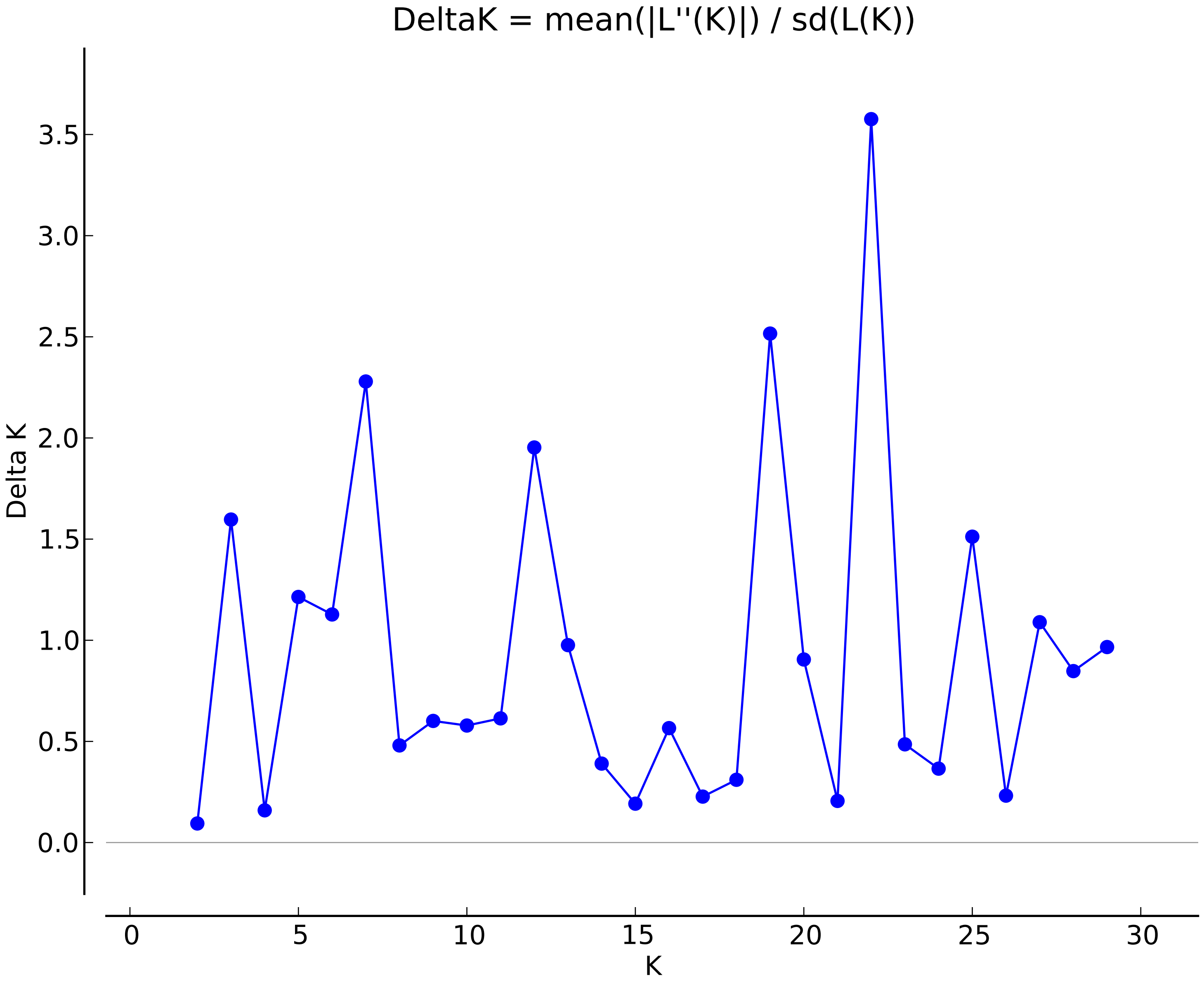

FIGURE S1e: Detection of the most likely number of genetically distinct groups in 2009.

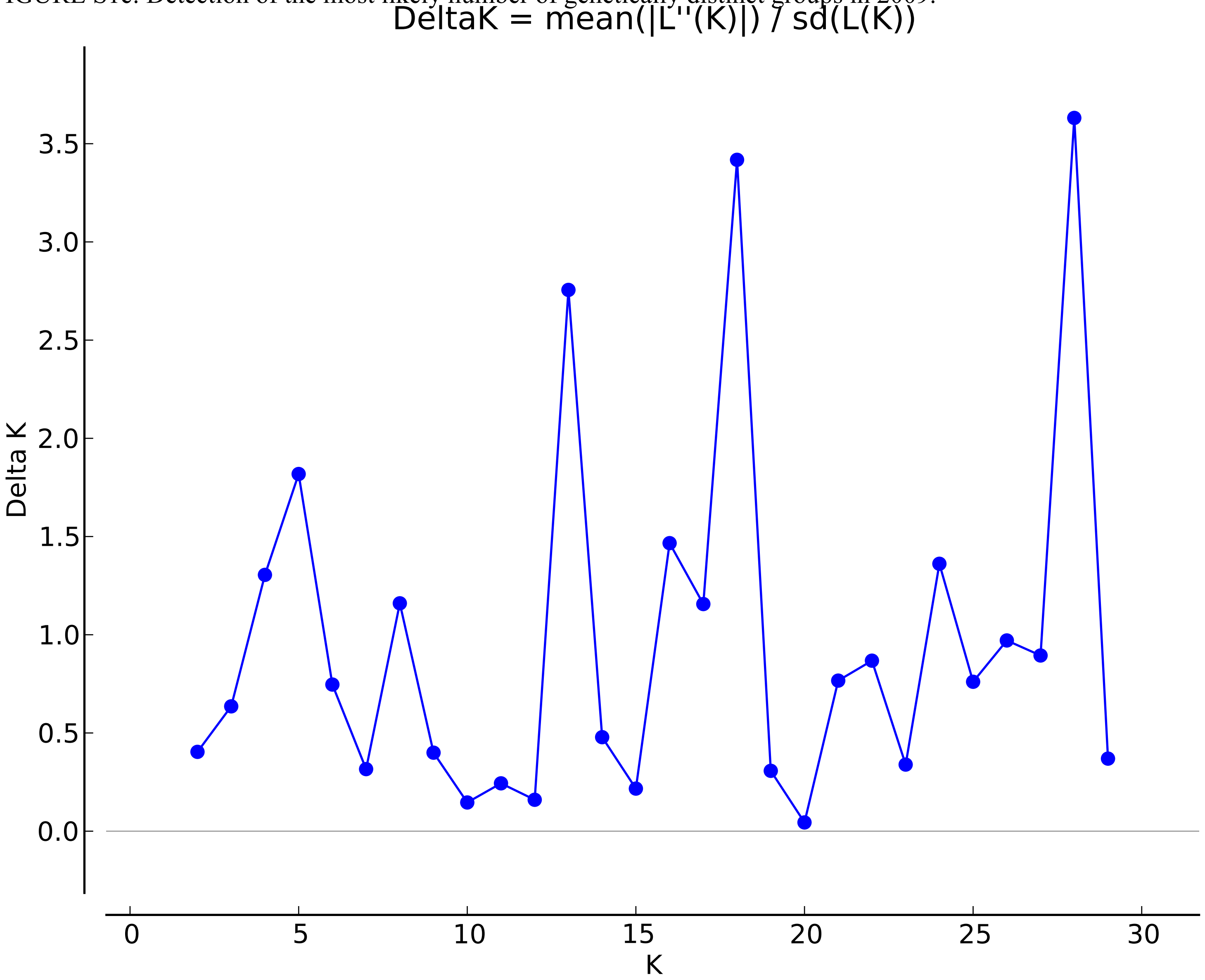

Supplement: Supplementary file 1 [file ECE3-7-7708-s001.pdf]
