# Supplementary material for: Genetic diversity and sex‐bias dispersal of plateau pika in Tibetan plateau
Source: Ecol Evol. 2017 Aug 22;7(19):7708–18. doi: 10.1002/ece3.3289 (PMC5632614; doi:10.1002/ece3.3289)
Supplement: Supplementary file 2 [file ECE3-7-7708-s002.docx]

Table S1: The information of individuals in the study.

|  | Sex | maturity | | | | Total | immaturity | | | | Total |
| --- | --- | --- | --- | --- | --- | --- | --- | --- | --- | --- | --- |
|  |  | May | June | July | August |  | May | June | July | August |  |
| 2005 | Female | 19 | 25 | 13 | 13 | 70 | 12 | 54 | 30 | 10 | 106 |
|  | Male | 9 | 16 | 7 | 9 | 41 | 8 | 48 | 23 | 11 | 90 |
|  | 307 | 28 | 41 | 20 | 22 | 111 | 30 | 102 | 53 | 21 | 196 |
| 2006 | Female | 5 | 2 | 1 | 0 | 8 | 15 | 45 | 19 | 38 | 125 |
|  | Male | 9 | 5 | 2 | 1 | 17 | 20 | 30 | 8 | 19 | 77 |
|  | 219 | 14 | 7 | 3 | 1 | 25 | 35 | 75 | 27 | 57 | 194 |
| 2007 | Female | 2 | 6 | 4 | 2 | 14 | 2 | 27 | 22 | 9 | 60 |
|  | Male | 11 | 4 | 1 | 2 | 18 | 4 | 27 | 23 | 14 | 68 |
|  | 160 | 13 | 10 | 5 | 4 | 42 | 6 | 54 | 45 | 23 | 128 |
| 2008 | Female | 13 | 15 | 14 | 8 | 50 | 18 | 114 | 30 | 13 | 175 |
|  | Male | 2 | 14 | 13 | 8 | 37 | 11 | 35 | 25 | 19 | 90 |
|  | 342 | 15 | 29 | 27 | 16 | 97 | 29 | 149 | 55 | 32 | 265 |
| 2009 | Female | 0 | 8 | 5 | 1 | 14 | 9 | 37 | 44 | 28 | 118 |
|  | Male | 11 | 14 | 7 | 7 | 39 | 9 | 47 | 45 | 43 | 144 |
|  | 315 | 11 | 22 | 12 | 8 | 53 | 18 | 84 | 89 | 71 | 262 |
| Total | 1343 |  |  |  |  |  |  |  |  |  |  |

Table S2: The sequence, repeat motif, Tm and fluorescence of primers

| GenBank Accession No. | Sequence (5-3) | Repeat motif | Annealing temp. | Dye |
| --- | --- | --- | --- | --- |
| EU518191 | GGGGCTGAAGACCATCTGAAA | (CA)_17_ | 62-60 | ROX |
|  | GTTCTTTACCTTCGGCCTCTGTGACT |  |  |  |
| EU518192 | TCTGATAAGGGCTCTCCAGC | (GT)_12_ | 61-59 | FAFM |
|  | GTTCTTTGAAGCCCCAGTGTAGCTCT |  |  |  |
| EU518193 | GAAATCGCAGCATCTCACAA | (TG)_16_(CG)_8_ | 61-59 | HEX |
|  | GTTCTTTCAAGCTTATCAATTGGGCTG |  |  |  |
| EU518194 | GCCAGGATGGGTCACTTAAA | (AC)_25_ | 58-56 | FAM |
|  | GTTCTTGACCTGCTGCCTTAGCTGTC |  |  |  |
| EU518196 | GCTCCCCTGACATCAGACAT | (GA)_24_ | 62-60 | HEX |
|  | GTTCTTACGCACGGGGAAAACTATG |  |  |  |
| EU518184 | TGGGAGACTTCTCTGTGCCT | (TAC)_8_(TAA)_4_ | 61-59 | ROX |
|  | GTTCTTGTGCCCTGTGCAGTGTCTTA |  |  |  |
| EU518185 | GACGTGGTCATCCAAGTCCT | (AC)_22_ | 58-56 | FAM |
|  | GTTCTTTTGCTGTGAAGTGTTCTCCCT |  |  |  |
| EU518186 | ACCAGCCCCTTGAAGTTTTT | (TC)_18_(TG)_11_tt(TG)_4_ta | 64-62 | FAM |
|  | GTTCTTTAGCCATCATCGCAAGTCTG |  |  |  |
| EU518187 | AATGCGAAAGTGAAATTCGG | GT/16 | 58-56 | HEX |
|  | GTTCTTTAAGTGGGACCACTAACGGC |  |  |  |
| EU518189 | CATTCTTGCCCTGTTGGTTCT | TGT/7 | 61-59 | FAM |
|  | GTTCTTTTCTACCCACCCATCACCAT |  |  |  |

Table S3: The gene diversity of each locus in five years

| 2005 | May | June | July | August |
| --- | --- | --- | --- | --- |
| Loc1 | 0.879 | 0.9 | 0.897 | 0.89 |
| Loc2 | 0.849 | 0.85 | 0.844 | 0.884 |
| Loc3 | 0.837 | 0.843 | 0.819 | 0.818 |
| Loc4 | 0.656 | 0.698 | 0.706 | 0.74 |
| Loc5 | 0.839 | 0.871 | 0.88 | 0.872 |
| Loc6 | 0.88 | 0.889 | 0.904 | 0.896 |
| Loc7 | 0.813 | 0.846 | 0.86 | 0.838 |
| Loc8 | 0.812 | 0.81 | 0.81 | 0.845 |
| Loc9 | 0.715 | 0.7 | 0.739 | 0.8 |
| Loc10 | 0.774 | 0.829 | 0.832 | 0.857 |
| Mean | 0.8054 | 0.8236 | 0.8291 | 0.844 |
|  |  |  |  |  |
| 2006 | May | June | July | August |
| Loc1 | 0.871 | 0.884 | 0.872 | 0.897 |
| Loc2 | 0.833 | 0.855 | 0.844 | 0.847 |
| Loc3 | 0.784 | 0.792 | 0.801 | 0.825 |
| Loc4 | 0.646 | 0.621 | 0.669 | 0.658 |
| Loc5 | 0.869 | 0.852 | 0.869 | 0.85 |
| Loc6 | 0.865 | 0.885 | 0.879 | 0.85 |
| Loc7 | 0.837 | 0.849 | 0.847 | 0.81 |
| Loc8 | 0.783 | 0.827 | 0.844 | 0.802 |
| Loc9 | 0.604 | 0.61 | 0.695 | 0.693 |
| Loc10 | 0.746 | 0.817 | 0.701 | 0.77 |
| Mean | 0.7838 | 0.7992 | 0.8021 | 0.8002 |
|  |  |  |  |  |
| 2007 | May | June | July | August |
| Loc1 | 0.937 | 0.891 | 0.894 | 0.837 |
| Loc2 | 0.866 | 0.854 | 0.872 | 0.945 |
| Loc3 | 0.808 | 0.888 | 0.821 | 0.868 |
| Loc4 | 0.671 | 0.684 | 0.743 | 0.655 |
| Loc5 | 0.877 | 0.874 | 0.883 | 0.854 |
| Loc6 | 0.892 | 0.886 | 0.898 | 0.873 |
| Loc7 | 0.853 | 0.835 | 0.86 | 0.83 |
| Loc8 | 0.806 | 0.837 | 0.793 | 0.792 |
| Loc9 | 0.852 | 0.796 | 0.798 | 0.692 |
| Loc10 | 0.779 | 0.883 | 0.783 | 0.815 |
| Mean | 0.8341 | 0.8428 | 0.84022 | 0.8161 |
|  |  |  |  |  |
| 2008 | May | June | July | August |
| Loc1 | 0.876 | 0.861 | 0.922 | 0.897 |
| Loc2 | 0.857 | 0.858 | 0.871 | 0.833 |
| Loc3 | 0.841 | 0.828 | 0.834 | 0.851 |
| Loc4 | 0.775 | 0.724 | 0.746 | 0.725 |
| Loc5 | 0.858 | 0.892 | 0.86 | 0.887 |
| Loc6 | 0.892 | 0.875 | 0.857 | 0.857 |
| Loc7 | 0.832 | 0.827 | 0.822 | 0.834 |
| Loc8 | 0.817 | 0.805 | 0.839 | 0.854 |
| Loc9 | 0.818 | 0.798 | 0.783 | 0.855 |
| Loc10 | 0.787 | 0.828 | 0.857 | 0.81 |
| Mean | 0.8353 | 0.8296 | 0.8391 | 0.8403 |
|  |  |  |  |  |
| 2009 | May | June | July | August |
| Loc1 | 0.897 | 0.903 | 0.896 | 0.91 |
| Loc2 | 0.874 | 0.869 | 0.841 | 0.861 |
| Loc3 | 0.844 | 0.863 | 0.863 | 0.843 |
| Loc4 | 0.717 | 0.711 | 0.711 | 0.713 |
| Loc5 | 0.897 | 0.882 | 0.881 | 0.893 |
| Loc6 | 0.901 | 0.884 | 0.878 | 0.872 |
| Loc7 | 0.868 | 0.863 | 0.869 | 0.864 |
| Loc8 | 0.842 | 0.835 | 0.814 | 0.831 |
| Loc9 | 0.78 | 0.821 | 0.754 | 0.77 |
| Loc10 | 0.754 | 0.806 | 0.838 | 0.758 |
| Mean | 0.8374 | 0.8437 | 0.8345 | 0.8315 |

Table S4: The allelic richness of each locus in five years

| 2005 | May | June | July | August | Mean |
| --- | --- | --- | --- | --- | --- |
| Loc1 | 14.088 | 13.317 | 14.151 | 11.608 | 13.474 |
| Loc2 | 9.349 | 8.8 | 8.65 | 8.999 | 8.892 |
| Loc3 | 11.498 | 11.403 | 10.57 | 7.822 | 10.976 |
| Loc4 | 5.417 | 6.679 | 6.216 | 6.516 | 6.378 |
| Loc5 | 10.484 | 11.998 | 11.64 | 13.236 | 12.022 |
| Loc6 | 11.129 | 13.576 | 15.857 | 12.271 | 13.835 |
| Loc7 | 7.967 | 11.85 | 11.449 | 10 | 11.172 |
| Loc8 | 7.206 | 6.805 | 6.386 | 7.806 | 6.97 |
| Loc9 | 9.081 | 9.319 | 10.874 | 7.904 | 9.857 |
| Loc10 | 15.646 | 14.953 | 15.247 | 13.931 | 15.148 |
|  |  |  |  |  |  |
| 2006 | May | June | July | August | Mean |
| Loc1 | 11.843 | 11.974 | 10.628 | 11.975 | 11.84 |
| Loc2 | 7.328 | 8.91 | 7.7 | 8.288 | 8.302 |
| Loc3 | 10.0069 | 9.396 | 6.928 | 10.393 | 9.575 |
| Loc4 | 4.399 | 4.158 | 3.932 | 4.746 | 4.386 |
| Loc5 | 11.241 | 10.921 | 10.62 | 10.317 | 10.789 |
| Loc6 | 9.825 | 10.511 | 10.624 | 10.43 | 10.756 |
| Loc7 | 8.134 | 8.707 | 8.596 | 6.391 | 8.34 |
| Loc8 | 5.578 | 6.678 | 7.828 | 6.815 | 6.809 |
| Loc9 | 7.54 | 9.853 | 7 | 8.007 | 8.986 |
| Loc10 | 10.939 | 13.803 | 11.583 | 11.708 | 12.865 |
|  |  |  |  |  |  |
| 2007 | May | June | July | August | Mean |
| Loc1 | 11.584 | 10.922 | 9.721 | 7.926 | 10.539 |
| Loc2 | 8.158 | 8.169 | 7.998 | 8.499 | 8.071 |
| Loc3 | 8.134 | 10.359 | 8.311 | 8.591 | 9.047 |
| Loc4 | 4.502 | 4.362 | 5.826 | 4.129 | 4.832 |
| Loc5 | 8.664 | 9.563 | 10.133 | 7.647 | 9.355 |
| Loc6 | 9.694 | 9.325 | 9.676 | 8.963 | 9.383 |
| Loc7 | 8.134 | 7.067 | 7.739 | 6.889 | 7.608 |
| Loc8 | 5.727 | 6.934 | 5.42 | 6.133 | 6.27 |
| Loc9 | 9 | 7.921 | 7.75 | 5.991 | 7.716 |
| Loc10 | 8.293 | 10.3 | 8.645 | 8.68 | 9.232 |
|  |  |  |  |  |  |
| 2008 | May | June | July | August | Mean |
| Loc1 | 12.044 | 13.431 | 16.034 | 16.453 | 14.534 |
| Loc2 | 9.628 | 8.701 | 9.361 | 8.731 | 8.924 |
| Loc3 | 10.565 | 11.076 | 10.284 | 12.05 | 11.331 |
| Loc4 | 5.718 | 6.124 | 6.319 | 6.299 | 6.227 |
| Loc5 | 10.515 | 11.213 | 12.267 | 12.964 | 11.641 |
| Loc6 | 11 | 10.673 | 10.181 | 9.635 | 10.43 |
| Loc7 | 8.86 | 8.947 | 7.756 | 7.887 | 8.778 |
| Loc8 | 7.848 | 8.096 | 9.169 | 9.735 | 8.888 |
| Loc9 | 11.433 | 11.757 | 12.564 | 12.271 | 12.367 |
| Loc10 | 16.298 | 15.572 | 15.379 | 14.352 | 15.418 |
|  |  |  |  |  |  |
| 2009 | May | June | July | August | Mean |
| Loc1 | 13.355 | 13.489 | 13.95 | 13.473 | 13.581 |
| Loc2 | 7.975 | 8.805 | 8.123 | 8.576 | 8.51 |
| Loc3 | 11.053 | 11.415 | 11.221 | 9.7 | 11.003 |
| Loc4 | 4.682 | 4.823 | 4.967 | 5.003 | 4.914 |
| Loc5 | 11.661 | 11.714 | 11.966 | 13.157 | 12.224 |
| Loc6 | 10 | 11.062 | 10.965 | 10.619 | 11.033 |
| Loc7 | 9.437 | 8.899 | 9.376 | 9.413 | 9.173 |
| Loc8 | 6.972 | 7.545 | 6.807 | 6.869 | 7.214 |
| Loc9 | 9 | 10.324 | 10.677 | 9.867 | 10.169 |
| Loc10 | 11.802 | 12.642 | 13.351 | 11.885 | 12.797 |

Table S5: The Fis of each locus in five years

| 2005 | May | June | July | August |
| --- | --- | --- | --- | --- |
| Loc1 | 0.129 | 0.058 | -0.098 | -0.07 |
| Loc2 | 0.151 | 0.132 | 0.099 | 0.337 |
| Loc3 | 0.123 | 0.09 | 0.023 | 0.105 |
| Loc4 | 0.064 | 0.095 | 0.159 | 0.309 |
| Loc5 | 0.085 | 0.223 | 0.144 | 0.363 |
| Loc6 | 0.581 | 0.036 | 0.04 | 0.02 |
| Loc7 | -0.035 | 0.212 | 0.108 | 0.082 |
| Loc8 | 0.342 | 0.158 | 0.14 | 0.283 |
| Loc9 | 0.651 | 0.543 | 0.571 | 0.594 |
| Loc10 | -0.1 | 0.098 | 0.094 | 0.067 |
|  |  |  |  |  |
| 2006 | May | June | July | August |
| Loc1 | 0.005 | 0.081 | 0.073 | 0.002 |
| Loc2 | 0.093 | 0.033 | 0.052 | 0.153 |
| Loc3 | 0.089 | -0.042 | 0.001 | 0 |
| Loc4 | -0.044 | -0.024 | 0.153 | 0.072 |
| Loc5 | -0.004 | 0.138 | 0.127 | 0.033 |
| Loc6 | 0.075 | 0.03 | 0.176 | 0.134 |
| Loc7 | 0.0213 | 0.097 | 0.056 | 0.022 |
| Loc8 | 0.227 | 0.131 | 0.309 | 0.114 |
| Loc9 | 0.474 | 0.379 | 0.738 | 0.556 |
| Loc10 | 0.152 | 0.098 | 0.236 | 0.079 |
|  |  |  |  |  |
| 2007 | May | June | July | August |
| Loc1 | 0.326 | 0.092 | 0.068 | 0.127 |
| Loc2 | -0.026 | 0.157 | 0.189 | 0.112 |
| Loc3 | 0.089 | 0.017 | 0.009 | 0.113 |
| Loc4 | -0.255 | 0.185 | 0.073 | -0.017 |
| Loc5 | 0.34 | 0.094 | 0.082 | -0.077 |
| Loc6 | -0.003 | 0.143 | 0.049 | 0.067 |
| Loc7 | 0.218 | 0.023 | 0.041 | -0.055 |
| Loc8 | 0.535 | 0.127 | 0.168 | 0.254 |
| Loc9 | 0.511 | 0.692 | 0.81 | 0.696 |
| Loc10 | 0.257 | 0.141 | 0.13 | 0.151 |
|  |  |  |  |  |
| 2008 | May | June | July | August |
| Loc1 | 0.097 | 0.042 | 0.147 | 0.009 |
| Loc2 | 0.061 | 0.023 | 0.049 | 0.026 |
| Loc3 | -0.02 | 0.042 | 0.057 | 0.118 |
| Loc4 | 0.041 | 0.139 | 0.107 | 0.047 |
| Loc5 | 0.106 | 0.103 | 0.051 | 0.172 |
| Loc6 | -0.001 | 0.092 | 0.116 | -0.058 |
| Loc7 | 0.118 | 0.089 | -0.112 | 0.226 |
| Loc8 | 0.147 | 0.306 | 0.509 | 0.305 |
| Loc9 | 0.549 | 0.601 | 0.405 | 0.6 |
| Loc10 | 0.023 | 0.14 | 0.185 | 0.225 |
|  |  |  |  |  |
| 2009 | May | June | July | August |
| Loc1 | 0.051 | 0.066 | 0.095 | 0.124 |
| Loc2 | -0.049 | 0.098 | 0.028 | 0.035 |
| Loc3 | 0.052 | 0.013 | 0.029 | 0.086 |
| Loc4 | -0.01 | 0.195 | 0.179 | 0.138 |
| Loc5 | 0.283 | 0.098 | 0.282 | 0.178 |
| Loc6 | 0.445 | 0.358 | 0.288 | 0.166 |
| Loc7 | 0.114 | 0.146 | 0.181 | 0.139 |
| Loc8 | 0.258 | 0.327 | 0.322 | 0.377 |
| Loc9 | 0.487 | 0.674 | 0.629 | 0.485 |
| Loc10 | 0.171 | 0.122 | 0.292 | 0.134 |

Table S6: The genetic variation characteristics of three pika species

| Species | Population size | Elevation | Mean no.alleles | FIS | Mean PIC | AR | Ho |
| --- | --- | --- | --- | --- | --- | --- | --- |
| American pikas^1^ | 74 | 1032-1254 m | **/** | 0.3 | **/** | 2.78 | 0.39 |
| Ochotona collaris ^2^ | 596 | 1800-2000 m | 6.33 | 0.001 | 0.577 | **/** | **/** |
| Plateau pikas | 1352 | 3846 m | 15.8 | 0.16 | 0.802 | 9.85 | 0.7 |

1. Data from Kelsey, Clayton, & Michael, 2016; 2. Data from Jessie & David, 2012
